# Epidemiology of Vector-Borne Diseases 2.0

**DOI:** 10.3390/microorganisms10081555

**Published:** 2022-08-01

**Authors:** Denis Sereno

**Affiliations:** Institut de Recherche pour le Développement, Université de Montpellier, UMR INTERTRYP IRD, CIRAD, Parasite Infectiology and Public Health Group, 34032 Montpellier, France; denis.sereno@ird.fr

Arthropods’ vectors—those of a large variety of families, including Culicidae, Simuliidae, Psychodidae, Ixodidae, Agarsidae, Pulicidae, Glossinidae, Reduviidae, and Tabanidae [1]—are vectors of viruses, bacteria, or parasitic pathogens, and are responsible for diseases which are devastating worldwide (dengue, viral encephalitis, bartonellosis, tularemia, lyme diseases, malaria, leishmaniasis, trypanosomiasis, filariasis, etc.). Over the past 30 years—following decades during which many vector-borne human illnesses were controlled in many areas through habitat modification and pesticides—malaria and dengue fever have reemerged in Asia and the Americas; West Nile virus has spread rapidly throughout the United States. Climate change—which has resulted in a modification of weather patterns and an increase in extreme events—affects vector-borne disease outbreaks [2], and is likely to have both short- and long-term effects on their transmission and infection patterns, affecting seasonal risk and broad geographic changes in disease occurrence. Models for predicting the effects of climate change on vector-borne diseases are subject to a high degree of uncertainty due to complex transmission cycles involving vectors, intermediate zoonotic hosts, and humans, in addition to the number of social and environmental drivers of vector-borne disease transmission. While climate variability and global change alter the transmission of vector-borne diseases, they also interact with pathogens adaptation, host availability, changes in ecosystems and land use, demography, human behavior, and adaptive capacity. Therefore, these impacts on the epidemiology and incidence of most vector-borne diseases—changing the vector and reservoir distribution—can allow unexpected contact between vector reservoirs and pathogens. In addition, the recent COVID-19 pandemic will have affected vector-borne disease transmission, incidence, and treatment, but such impacts are poorly documented [3,4]. To improve the surveyal and detection of these diseases, a key point is the survey of a limited number of arthropod vectors whose identification rely on highly skilled individuals or on costly material or infrastructure. In addition, it is only the detection of pathogenic micro-organisms (viruses, bacteria, parasites, etc.) that allows for the warning of a potential epidemy. Therefore, the real-time monitoring of hematophagous insects (such as mosquitoes) in the field and the identification of pathogens they carry is a challenge for foreseeing vaccination campaigns and restraining the potential spreading of diseases.

A renewal in the epidemiological data on vector-borne diseases is now needed, to update the current information on the impact of these diseases worldwide and to foresee endemic, emerging, or re-emerging ones. Hence, information is needed surrounding the possible influences of climate change and/or the COVID-19 pandemic on vectors and hosts distributions and dynamics, disease incidence and epidemiology, and the underlying factors which trigger vector-borne transmission. Furthermore, these factors influence the possible spread of diseases from endemic to non-endemic areas. In addition, methodologies and models that support the field surveyal of arthropod vectors and the pathogens they transmit are required.

The current Special Issue, entitled “Epidemiology of Vector-Borne Diseases 2.0”, intends to provide a framework for (1) the analyses of transmission risks and (2) developing future solutions to fight vector-borne diseases, including neglected tropical diseases. This Special Issue will also provide updated knowledge on vector-borne disease transmission, in support of the management of these diseases within the public health sector.
